# Idiosyncratic fixation patterns generalize across dynamic and static facial expression recognition

**DOI:** 10.1038/s41598-024-66619-4

**Published:** 2024-07-13

**Authors:** Anita Paparelli, Nayla Sokhn, Lisa Stacchi, Antoine Coutrot, Anne-Raphaëlle Richoz, Roberto Caldara

**Affiliations:** 1 Eye and Brain Mapping Laboratory (iBMLab), Department of Psychology, University of Fribourg, Faucigny 2, 1700 Fribourg, Switzerland; 2 Laboratoire d’Informatique en Image Et Systèmes d’information, French Centre National de La Recherche Scientifique, University of Lyon, Lyon, France

**Keywords:** Individual differences – facial expressions of emotion – eye-movements, Emotion, Social behaviour

## Abstract

Facial expression recognition (FER) is crucial for understanding the emotional state of others during human social interactions. It has been assumed that humans share universal visual sampling strategies to achieve this task. However, recent studies in face identification have revealed striking idiosyncratic fixation patterns, questioning the universality of face processing. More importantly, very little is known about whether such idiosyncrasies extend to the biological relevant recognition of static and dynamic facial expressions of emotion (FEEs). To clarify this issue, we tracked observers’ eye movements categorizing static and ecologically valid dynamic faces displaying the six basic FEEs, all normalized for time presentation (1 s), contrast and global luminance across exposure time. We then used robust data-driven analyses combining statistical fixation maps with hidden Markov Models to explore eye-movements across FEEs and stimulus modalities. Our data revealed three spatially and temporally distinct equally occurring face scanning strategies during FER. Crucially, such visual sampling strategies were mostly comparably effective in FER and highly consistent across FEEs and modalities. Our findings show that spatiotemporal idiosyncratic gaze strategies also occur for the biologically relevant recognition of FEEs, further questioning the universality of FER and, more generally, face processing.

## Introduction

Facial expression recognition (FER) is an essential skill for human social interactions. Faces convey an extensive array of socio-emotional information about one individual from which this person’s emotional state can be inferred, enabling a highly adaptive behavior in the observer. The existence of six “universal emotional expressions”: anger, disgust, fear, happiness, sadness, and surprise, has been widely accepted in the field^1,2^. Supportive of Darwin’s pioneering work^3^ these basic internal states were claimed to be hard-wired by virtue of their evolutionary roots, and therefore communicated and recognized equally by humans. Owing to this view, traditional research in FER assumed that humans universally read facial expressions of emotion (FEEs) by using similar visual strategies. Yet, during the past decade, this vision has been amply challenged by cross-sectional and eye-movement studies in face perception that have demonstrated important attentional differences between cultures and individuals (for a review see^4–6^).

Cultural findings in various face categorization tasks have challenged the notion of universality, contributing to the ongoing “nature versus nurture” debate. During face recognition, studies revealed distinct visual strategies employed by Western Caucasians and East Asians observers. Westerners tend to focus their fixations predominantly to the eye region, while Easterners direct their attention towards the central area of the face^7–9^. Similarly, these cultural differences have also been observed in emotion recognition tasks^10–13^. Jack and colleagues^12^ were the first to report cultural differences in eye movements during FER. Easterners consistently fixated the eyes compared to Westerners, which were focusing on the mouth. A subsequent study using psychophysical reverse correlation techniques confirmed and refined this observation^14^. With the use of those methods, Jack and colleagues^14^ showed cultural specificities in the way FEEs are internally represented, further endorsing the view that Easterners code FEEs based on information coming from the eye region, whereas Westerners rely more heavily on cues from the mouth region. These culturally-rooted fixation preferences emerge early in infancy, as demonstrated by a study showing that 7-month-old infants display fixation strategies that resemble those used by the adults of their cultural group^15^. Altogether, these findings highlight the significant role of culture in shaping the development of perceptual strategies for FER. Sensibly, these discrepancies extend beyond the simple contrast between Western versus Eastern populations^13,16^. When investigating social cognition across 12 Western countries, Quesque and colleagues^16^ showed that nationality accounted for more than 20% of the variance observed in FER scores. Moreover, other factors such as age, gender, and education also showed to have an impact on FER. Altogether, these findings emphasize human diversity for FER and the need to clarify the extent to which FER is rooted or not in similar processes even within a single culture.

Studies investigating idiosyncratic eye-movements during face recognition have shown that individuals deviate from the widely-accepted T-shaped scanning pattern^17–20^ (primarily focusing on the eyes and nose) which has been consistently reported in Western observers. In fact, this T-shaped scanning pattern reflects an average across all observers, masking individual differences in fixation patterns. Such averaging process leads most psychological theories to be based on these aggregating artefacts^18,19^, which are often not representative of individual observers. Adding to these findings, a recent study in face recognition demonstrated fixations towards the preferred idiosyncratic facial features were robustly linked to stronger neural face identity discrimination response^17^. Others have reported how distinct individual strategies in perceptual tasks achieve comparable behavioral performance^18,21^. Altogether, these findings refute the concept of a single face representation format shared across observers and posit individual differences as a valuable source of *signal* rather than *noise*. Whitin this framework, to date, only a single eye-tracking study from Yitzhak and colleagues^21^ has investigated individual differences in visual strategies during the decoding of dynamic FEEs. These authors divided the face into regions of interest (ROI)s and showed that observers varied in their facial feature fixation preference (eyes, nose, and mouth regions) during FER, while achieving comparable performance. However, there is an important long-standing debate regarding the validity of ROIs analyses in eye-tracking research. With this approach, visual inputs to the face are segmented and bounded to chosen feature location (i.e.: eye region vs. mouth region), but this division is -by definition- constrained by subjective evaluations, it is not precise, difficult to reproduce and is therefore scientifically problematic. This factor alone contributes to the great variability observed in eye-movement findings. Furthermore, participants in perception tasks can be erroneously placed into one single face region group based on the most quantitative time they spend on it, when perhaps they are looking at two different regions with statistically comparable importance. Data-driven methods can be employed to overcome those important methodological shortcomings. In our study, we thus combined two robust unbiased data-driven methods computing statistical fixation maps of eye movements instead of applying the subjective segmentation of the experimental image used as a stimulus into given ROIs. We isolated idiosyncratic scanning profiles with two toolboxes: the EMHMM^22^ and iMap4^23^. EMHMM^22^ captures precise individual differences by incorporating both spatial and temporal information, ultimately shunting a clear-cut face pattern result for varying clusters. iMap 4^23^ computes robust statistical fixation analyses across tasks, by applying a Gaussian kernel on each eye fixation to generate smooth fixation patterns and average them. Both data-driven methods are extremely well-suited to isolate statistical differences in eye-movement behavior.

In addition, Yitzhak and colleagues^21^ used only dynamic FEEs that were unnaturally lasting for 6–9 s. This methodological choice both questions the validity of those observations and undermines the generalization of those previous findings to static faces^14,24–26^. Given those shortcomings, the testing of both static and ecologically valid dynamic FER is an important methodological control, as it is still debated whether the decoding of these modalities is fully comparable^24,26–28^ or if they relate to partially distinct neurofunctional routes^29,30^. For this purpose, we incorporated static stimuli into the experiment to test the generalization of idiosyncrasies across stimulus modalities, feeding further theories of FER. Secondly, we also aimed at mimicking the most natural duration of expressions in real-life^31,32^ and thus chose the FEE database created by Gold and colleagues^28^, which is composed of significantly shorter dynamic stimuli (lasting 1 s). This short duration controls for the recording and analysis of spurious eye movements potentially occurring after emotion identification.

To anticipate our findings, our data-driven statistical analyses revealed that Western observers show three distinct equally occurring fixation patterns to decode the six basic FEEs, which vary in the spatial and temporal dimensions. Notably, these gaze strategies exhibit high consistency across expressions and modalities with a few exceptions, and result in a comparable recognition performance in most cases. The visual information intake is not unique even for the biologically relevant recognition of facial expressions of emotion.

## Results

### Eye-movements profiles during facial expression recognition

**Figure 1:**
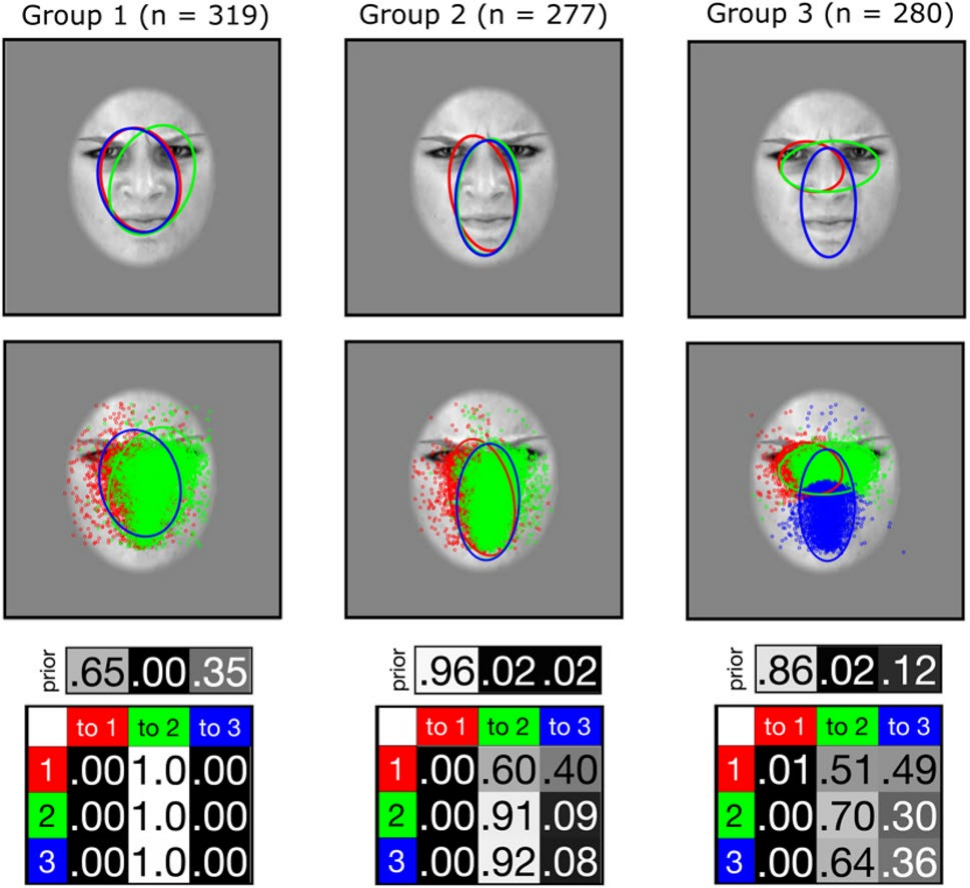
Fixation patterns (n = 3) in FER discovered through EMHMM clustering. Each representative HMM included three different states (k = 3), depicted by the sROI 1 (red), 2 (green) and 3 (blue). Please note that, as sROI 1 and 3 in Group 1, and sROI 2 and 3 in Group 2, were duplicates, an ellipse displacement of 1 pixel to the right of the figure was made for better visualization. Third row shows priors and transitions matrices. Priors represent the probability of the first fixation to belong to each state. Gaze transition probabilities between the three different states indicate the probabilities of observing a particular transition from one state to another, or to remain in the same state.

**Figure 2:**
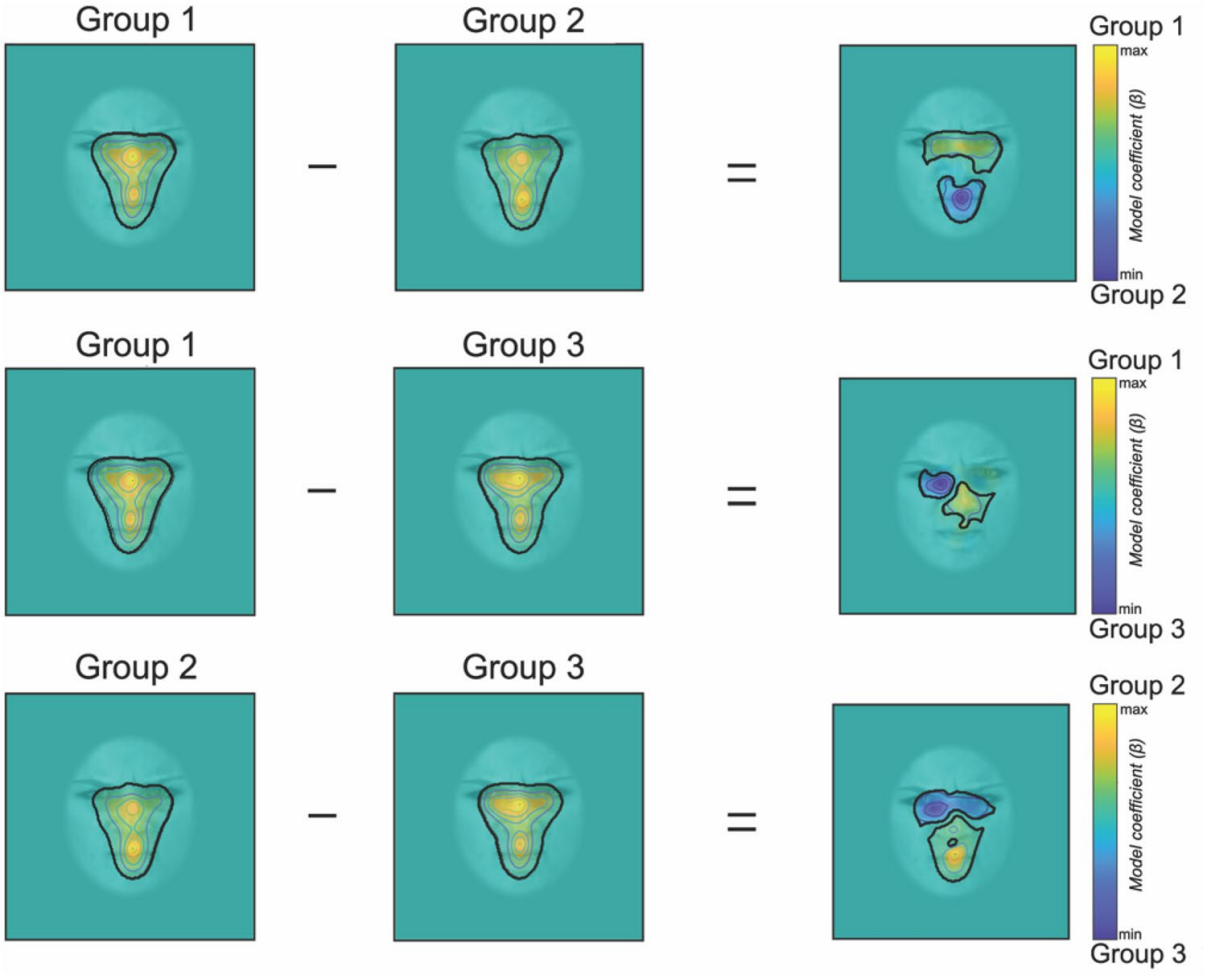
Heat maps illustrating the fixation bias of Group 1, 2 and 3, with their associated statistical difference. Significant areas are demarked by a black line. Yellow and blue clusters represent the respective groups’ differences.

The current experiment aimed to isolate the individual sampling strategies deployed by the observers during the recognition of the 6 basic facial FEEs in 2 modalities (i.e., static and dynamic). We first analyzed the fixations patterns of our participants with the EMHMM toolbox. EMHMM uses Hidden Markov Models (HMMs) to model individual eye-movements (EM) dataset while considering both person-specific regions of interest (ROIs) and transitions among the ROIs**.** Importantly, this analysis considered as input 12 EM datasets (6 FEEs × 2 modalities) per subject, for a total of 876 EM datasets. The algorithms that extracted individual as well as representative HMMs were blind to which dataset belonged to which condition or participants. This information was retrieved only in a second stage to assess whether the clustering procedure was FEE- or modality-dependent. The initial individual HMM obtained for each subject revealed that the median number of statistical ROIs (sROIs) that best described EM patterns was three. This was therefore the number of hidden states used during the subsequent clustering analysis. This procedure revealed that the 876 EM datasets could be clustered in three representative and significantly different fixation patterns. Specifically, a one-way ANOVA confirmed that data from Group 1 were more likely generated by the representative HMM of Group 1 rather than by those of Group 2 and 3 (F(2, 318) = 95.71, p < 0.001). The same pattern of results were obtained for data from Group 2 (F(2, 276) = 166.97, p < 0.001) and from Group 3 (F(2, 279) = 319.16, p < 0.001). A 3-sample test for equality of proportions without continuity correction revealed that Group 1 (319 participants), Group 2 (277 participants) and Group 3 (280 participants) were statistically comparable in size (χ^2^(2) = 5.64, p = 0.06). However, sROIs 1 and 3 and 2 and 3, in Group 1 and 2 respectively, are duplicate of each other’s (Supplementary Fig. 1). Therefore, their transition probabilities can be collapsed together. The final number of sROIs was nonetheless kept to three to fully account for the data of Group 3. The final three representative fixation patterns are illustrated in Figs. 1 and 2. Group 1’s first fixation was located within an area encompassing the mouth, left eye, and the internal corner of the right eye (after collapsing red and blue sROIs, 100% of cumulative starting probability – SP). Following fixations were then directed towards the region spanning between the mouth, nose, and right eye (green sROI, 100% transition probability -TP). Comparatively, the majority of Group 2’s first fixations were directed more towards the mouth, and only partially towards the left eye (red sROI, 96% SP). Subsequent fixations were then restricted within a central region of the face (after collapsing green and blue sROIs, 100% cumulative TP) spanning predominantly from the nasion to the mouth. The third Group discovered using EMHMM exhibits a more focal pattern. After directing the majority of their first fixation towards the left eye (red sROI, 86% SP), fixations shifted with similar probability towards either the midline of the face (blue sROI, 49% TP) or remained focused around the eyes (green sROI, 51% TP). In both cases, the next most likely fixation location was within the eye region (from midline to eye-region, 64% TP; no shift from the eye-region, 70% TP), while only a smaller proportion of fixations were redirected towards the face midline (from eye-region to midline, 30% TP; or no shift from midline, 36% TP).

### Spatial comparisons of fixation patterns with iMap4

To better disentangle the differences between the three representative fixation patterns discovered using EMHMM, we explored their spatial distributions using iMap4. iMap4 is a data-driven method that assesses statistical differences in terms of fixation distributions only, without considering their temporal relationship. Our linear mixed model revealed significant differences in the fixation location across the three Groups (Fig. 2). Specifically, the comparison of Group 1 and 2 revealed two significant clusters. The first cluster was characterized by a greater number of fixations towards the eye-region by Group 1 (F(1,128) = 63.24 at the local maximum, beta contrast = 1.37, 95% CI [1.03, 1.71], F(1,128) = 3.93 at the local minimum, beta contrast = 0.16, 95% CI [0.00, 0.32]). The second significant cluster was driven by Group 2 exhibiting a stronger fixation bias towards the mouth (F(1,128) = 106.05 at the local maximum, beta contrast = − 2.78, 95% CI [− 3.32, − 2.25]; F(1,128) = 3.92 at the local minimum, beta contrast = − 0.15, 95% CI [− 0.31 − 0.00]). Similarly, the comparison of Group 1 and 3 also revealed the presence of two significant clusters. Compared to Group 3, Group 1 fixated more often the nose region (F(1,128) = 36.65 at the local maximum, beta contrast = 0.71 95%, CI [0.48, 0.94], and F(1,128) = 3.91 at the local minimum, beta contrast = 0.29, 95% CI [0.00, 0.58]). On the other hand, Group 3 directed comparatively more fixations towards the left eye (F(1,128) = 49.52 at the local maximum, beta contrast = -1.93, 95% CI [− 2.47, − 1.39] and a F(1,128) = 3.91 local minimum, beta contrast = -0.31, 95% CI [− 0.62, 0.00]). Finally, the comparison between Group 2 and 3 also revealed two significant clusters. While Group 3 fixated more often the eye region (F(1,128) = 103.03 local maximum, beta contrast = − 2.67, 95% CI [− 3.18, − 2.14] and a F(1,128) = 3.92 local minimum, beta contrast = -0.40, 95% CI [− 0.80, 0.00]), Group 2 directed more fixations towards the mouth region (F(1,128) = 108.32 at the local maximum, beta contrast = 3.45, 95% CI [2.79, 0.94], and F(1,128) = 3.92 at the local minimum, beta contrast = 0.57, 95% CI [0.00, 1.14]).

### Generalization of sampling strategies across FEEs and modalities

**Figure 3:**
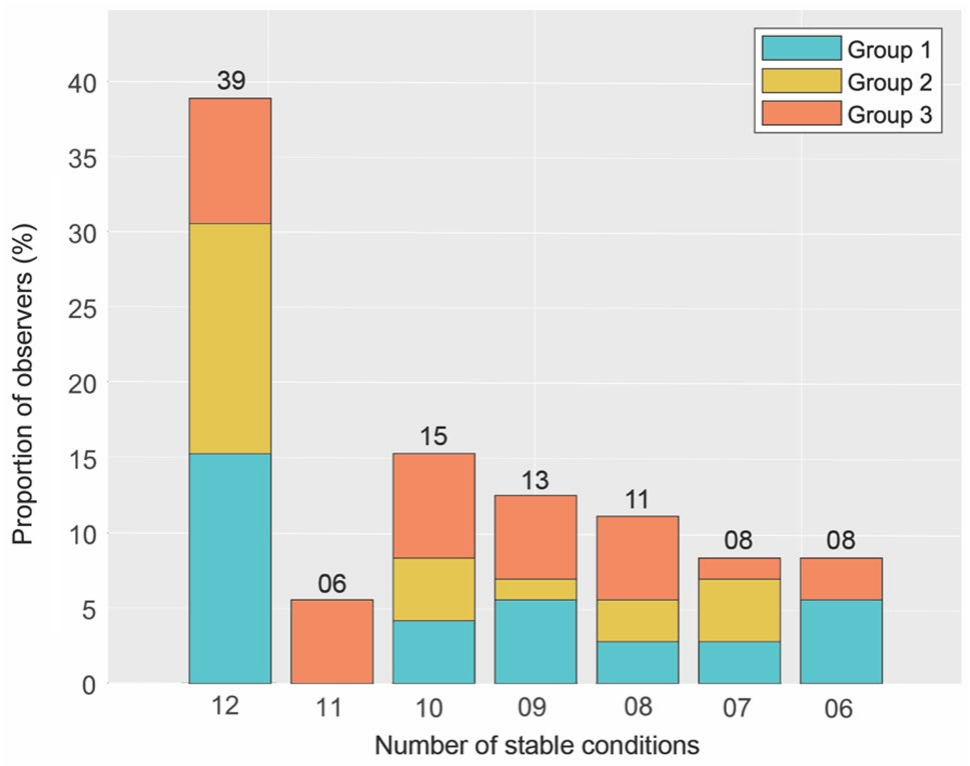
Distribution of 72 participants to their assigned prevalent groups of fixation strategy and their general level of consistency across the twelve eye-movement datasets. One subject was not included as their EM datasets were split over different groups without any prevailing over the others. Please note that 60% of the participants (“consistent observers”) use the same strategy for at least 10 out of 12 conditions.

EMHMM revealed three representative visual sampling strategies during FER, relating to specific fixation patterns. Each eye-movement dataset (73 subjects × 6 emotions × 2 modalities) was classified by EMHMM as belonging to one of these visual sampling patterns. In this section, we explored whether the clustering of these datasets was influenced by FEE or modality. In other words, for each subject, we determined whether different FEEs and stimulus modalities would trigger comparable or different sampling strategies. When considering the distribution of EM datasets for each observer across the three groups (Fig. 3), we found 28 out of 73 subjects (39%) to be “consistent observers”. Specifically, the eye-movement patterns exhibited across the 12 different conditions were all clustered within the same group, although different subject could belong to different groups. The proportion of consistent subjects was significantly higher compared to the proportion of observers consistent in only 6, 7, 8, 9, 10 or 11 conditions (χ^2^(1) = 20.63, p < 0.001; χ^2^(1) = 9.33, p < 0.01; χ^2^(1) = 11.33, p < 0.001; χ^2^(1) = 13.60, p < 0.001; χ^2^(1) = 17.55, p < 0.001; χ^2^(1) = 17.55, p < 0.001). Importantly, consistent participants were equally present across the three groups (χ^2^(2) = 1.79, p = 0.41). To account for the large number of conditions and therefore for the increased probability of finding one or two conditions clustered in a separate group by chance, we redefined the concept of a “consistent observer” to include those participants who showed a *stable* fixation strategy across at least 10 conditions. Forty-three subjects (60%) fit this definition and the subsequent comparison with the number of participants not meeting this criterion (30 individuals, 40%) showed a significant difference (χ^2^(1) = 4.99, p < 0.05). In the next section, we explore in more details the presence of sampling strategy’s (in)dependency on FEE and modality.

### Eye-movement patterns across modalities

**Figure 4:**
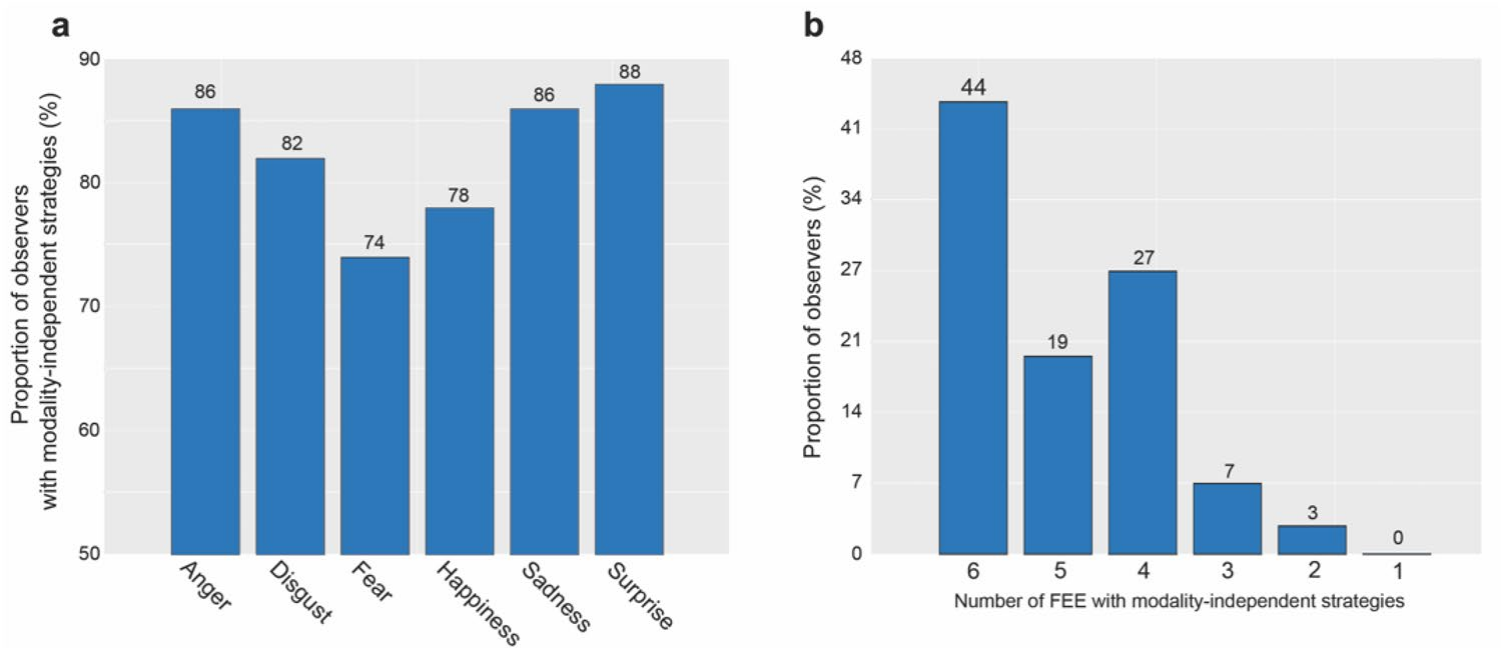
(**a**) Percentages of observers employing the same strategy (1, 2 or 3) for static and dynamic modalities for each expression. (**b**) Distribution of observers employing the same strategy (1, 2 or 3) for static and dynamic modalities, from 1 to 6 FEEs.

We first explored the impact of modality on eye-movements patterns for each FEE separately. Results showed that during recognition of anger, fear, sadness and surprise, the eye-movement patterns for static and dynamic stimuli were clustered within the same group for 86%, 82%, 86% and 88% of observers respectively (Fig. 4a). Sampling strategies were stable across modalities also for the recognition of disgust and happiness, although for a smaller proportion of subjects (74% and 78% respectively). These proportion differences between the 6 emotions were not significant (χ^2^(5) = 7.39, p = 0.19). Further examination revealed that 44% of the observers had stable fixation strategies across modalities for all emotions (Fig. 4b). It is however important to note that the emotions could differ in the eye-movement patterns they triggered and be therefore clustered in different groups (e.g., the EM patterns of participant X for anger in both static and dynamic conditions might be clustered within Group 1, while for the same participant the EM patterns for disgust, both static and dynamic, might be clustered within Group 2). The sampling strategies of the remaining observers (56%) were impacted by presentation modality to different degrees and depending on the FEE considered. Specifically, 26% of observers exhibited fixation patterns that were modality-independent for 5 emotions, 19% for 4 emotions, 5% for 3 emotions, 4% for 2 emotions and 1% for 1 emotion only (Fig. 4b). Differences in these proportions were statistically significant (χ^2^(5) = 75.05, p < 0.001). More precisely, the observers with modality-independent sampling strategies for all FEEs were significantly more than those who were stable for only 3, 2 or 1 emotion. This was also the case for observers who were stable for 5 FEEs compared to those who were stable for 1, 3 (p < 0.001) and 2 (p < 0.05) FEEs. The proportion of stable observers for 3 FEEs was significantly greater than the proportion of stable individuals for 2 (p < 0.05) or 1 (p < 0.001) FEE. Finally, a significantly higher proportion of observers were stable for 2 FEEs compared to those who were stable for only 1 FEE (p < 0.05). Importantly, all pairwise comparisons were corrected for multiple comparisons using the Holm-Bonferroni procedure.

### Eye-movement patterns across expressions

**Figure 5:**
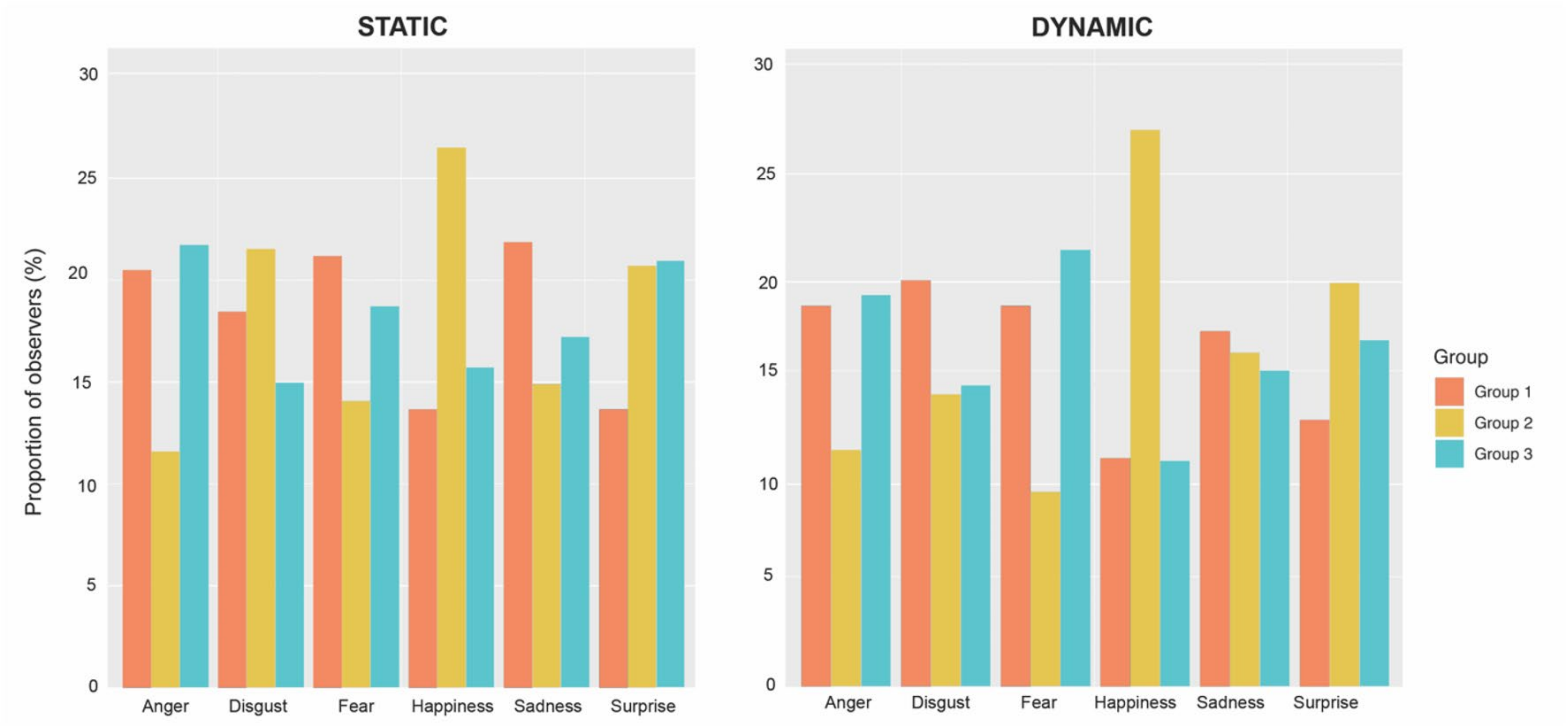
Distribution of EM datasets for all expressions *within* groups, respectively in static and dynamic modalities. Note that the distribution of expressions *between* groups is not represented here.

In this section, we explored whether a given facial expression of emotion would trigger a specific sampling strategy (Fig. 5). Comparing the presence of EM datasets associated to specific FEE across groups revealed that the sampling strategies deployed during the recognition of dynamic fear and static and dynamic happiness were more often clustered within Group 2 (dynamic fear: χ^2^(2) = 8.07, p < 0.01; static happiness: χ^2^(2) = 8.00, p < 0.05; dynamic happiness: χ^2^(2) = 18.61, p < 0.001). Specifically, sampling strategies associated with dynamic fear were significantly more present in Group 2 compared to Group 3 (p < 0.05), while the difference with Group 1 was not significant. Eye-movement pattern triggered by static happiness were significantly more present in Group 2 than Group 1 (p < 0.05) but not Group 3. Finally, dynamic happiness triggered sampling strategies that were more often clustered in Group 2 than Group 1 or 3 (p < 0.001). No other combination of FEE and modality of presentation showed a clear association with a specific group of eye-movement patterns. Finally, exploring the composition of each group, separately for each modality, revealed that the EM datasets associated to each emotion were equally frequent within Group 1(static: χ^2^(5) = 6.63, p = 0.24; dynamic: χ^2^(5) = 7.22, p = 0.20) and within Group 2 (static: χ^2^(5) = 3.32, p = 0.65; dynamic: χ^2^(5) = 6.41, p = 0.26). In contrast, within Group 3 we found a significantly different frequency of datasets associated with different emotions in both modalities (static: χ^2^(5) = 12.54, p < 0.05; dynamic: χ^2^(5) = 21.79, p < 0.001). Specifically, within the dynamic modality, EM datasets related to the recognition of happiness were significantly more present than those related to the recognition of anger (p < 0.05) and of fear (p < 0.01). Within the static modality, no contrasts were significant. Importantly, all pairwise comparisons were corrected using the Holm-Bonferroni procedure.

### Idiosyncratic fixation patterns and performance during FER

**Figure 6:**
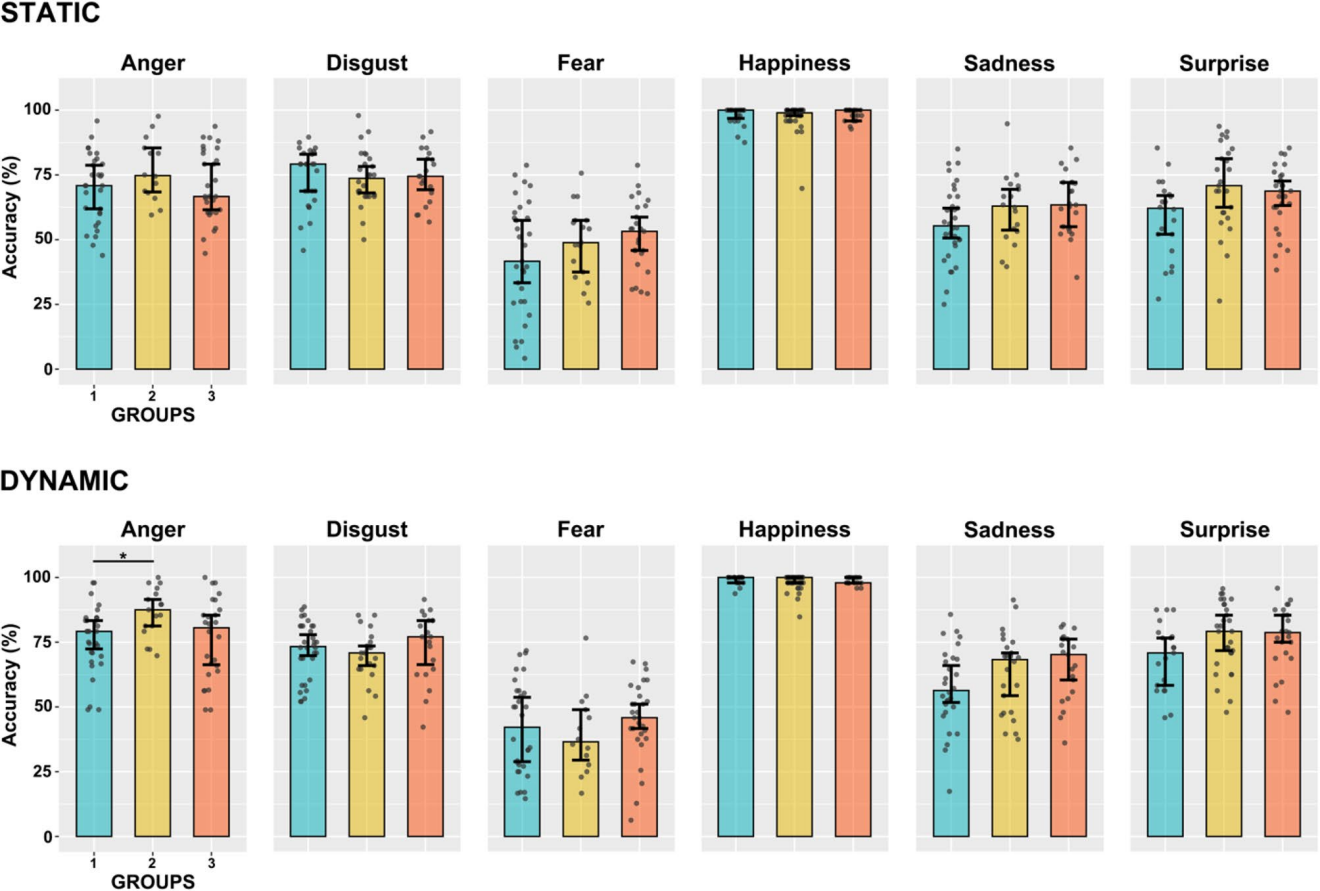
Observers’ FER accuracy in each of the twelve conditions across the three strategies. * p < 0.0017. Error bars represent 95% confidence intervals of the median number of correct responses for each group and condition.

In this final section, we investigated whether the three representative sampling strategies identified using EMHMM had any impact on facial expression recognition performance. Given the differences in our sample sizes and potential different variance across groups we carried out our analysis using the non-parametric Mann Whitney U-test. After retrieving the recognition accuracy related to each eye-movement dataset, we compared performance across groups for each emotion and modality independently. Applying Bonferroni correction for multiple comparisons, we only found a significant result for the recognition of the dynamic expression of anger (Fig. 6). Specifically, the recognition accuracy for the dynamic expression of anger was significantly higher in Group 2 compared to Group 1 (W(1) = 129,5, p < 0.01, 95% CI[-0.169, -0.037]). All other statistical values are included in Table 1.

**Table 1:** Group accuracy in FER Group accuracy in FER.

	Condition	Comparisons	W	p_val
1	Dynamique_Anger	Grp1—Grp2	129,5	* 0,00,562
2	Dynamique_Anger	Grp1—Grp3	384	0,92,798
3	Dynamique_Anger	Grp2—Grp3	306	0,03,576
4	Statique_Anger	Grp1—Grp2	155,5	0,17,331
5	Statique_Anger	Grp1—Grp3	426	0,8974
6	Statique_Anger	Grp2—Grp3	262	0,12,925
7	Dynamique_Disgust	Grp1—Grp2	386,5	0,36,241
8	Dynamique_Disgust	Grp1—Grp3	275	0,402
9	Dynamique_Disgust	Grp2—Grp3	159,5	0,19,187
10	Statique_Disgust	Grp1—Grp2	386	0,53,877
11	Statique_Disgust	Grp1—Grp3	281	0,8211
12	Statique_Disgust	Grp2—Grp3	250,5	0,84,185
13	Dynamique_Fear	Grp1—Grp2	230,5	0,61,413
14	Dynamique_Fear	Grp1—Grp3	390	0,49,974
15	Dynamique_Fear	Grp2—Grp3	144	0,12,934
16	Statique_Fear	Grp1—Grp2	230,5	0,48,346
17	Statique_Fear	Grp1—Grp3	305,5	0,1791
18	Statique_Fear	Grp2—Grp3	199	0,7389
19	Dynamique_Happinness	Grp1—Grp2	405	0,39,504
20	Dynamique_Happinness	Grp1—Grp3	163,5	0,2563
21	Dynamique_Happinness	Grp2—Grp3	321,5	0,66,304
22	Statique_Happinness	Grp1—Grp2	332,5	0,80,559
23	Statique_Happinness	Grp1—Grp3	213	0,94,263
24	Statique_Happinness	Grp2—Grp3	327	0,86,762
25	Dynamique_Sadness	Grp1—Grp2	266,5	0,20,528
26	Dynamique_Sadness	Grp1—Grp3	200	0,05,887
27	Dynamique_Sadness	Grp2—Grp3	215	0,40,625
28	Statique_Sadness	Grp1—Grp2	225,5	0,21,009
29	Statique_Sadness	Grp1—Grp3	249	0,04,313
30	Statique_Sadness	Grp2—Grp3	185,5	0,58,107
31	Dynamique_Surprise	Grp1—Grp2	193	0,02,899
32	Dynamique_Surprise	Grp1—Grp3	155	0,04,302
33	Dynamique_Surprise	Grp2—Grp3	349,5	0,77,508
34	Statique_Surprise	Grp1—Grp2	146,5	0,01,858
35	Statique_Surprise	Grp1—Grp3	189,5	0,05,967
36	Statique_Surprise	Grp2—Grp3	410	0,28,869

## Discussion

Our overarching goal was to investigate whether the eye movements exhibited during FER could be categorized into a unique or distinct groups of fixation patterns, isolate potential differences in sampling strategies, and assess the consistency of the observers’ eye-movements across stimulus modality (i.e., static *vs.* dynamic). We identified three distinct, equally effective, and mutually exclusive idiosyncratic visual sampling strategies for static and dynamic FER in Western observers. The first strategy involved vertical eye fixations alternating between two regions: one included the left eye, the other the right eye, and both included the mouth. The second strategy was characterized by eye movements that alternated less between individual face features and were more bound within the central region of the face. This included the inner corner of the eyes, the nose, and the whole mouth region. Finally, the third strategy markedly differed from the previous two from a temporal and a spatial point of view. Within this group, fixations alternated between the eye and a vertical area encompassing the nose and mouth regions. All three strategies were highly consistent across all six basic FEEs, modalities and, importantly, did not modulate FER performance. It is worth noting that these visual sampling strategies were identified by considering the fixation patterns resulting from 12 eye-movement (EM) datasets (observations for the 6 FEEs × 2 stimulus modalities), rather than categorizing observers by their sampling strategy averaged across 12 conditions. Our results showed that these visual fixation patterns were statistically stable. Most of the observers (60%) either predominantly favored one strategy among the three identified or adopted a non-systematic alternative strategy for only one or two conditions of the experimental task. Altogether, our findings refine previous research in emotion processing^21,33^ by showing *spatiotemporal* idiosyncratic differences in FER, strongly challenging the traditional view of a single face processing format for the decoding of FEE. Importantly, effective FER can be achieved by sampling different combinations of multiple facial features.

Yitzhak and colleagues^21^ previously addressed a similar question by using analyses on single predetermined face-feature (i.e., eye, nose, and mouth lookers), while excluding fixation transition across face regions. Additionally, their study was limited to the use of non-ecologically valid dynamic FEEs. To overcome these limitations, we used static and dynamic FEEs and combined two robust well-established data-driven eye-movement statistical approaches previously validated in other face perception studies^34–37^: the EMHMM^22^ and iMap4^23^ toolboxes. As a result, our data-driven statistical approaches specifically isolated the idiosyncratic *spatiotemporal* information of the fixations dedicated to the decoding of static and dynamic FEEs. More precisely, Group 1 displayed scan paths starting from two overlapping vertical left-central *statistical* Regions of Interest (sROIs) encircling the left eye and mouth. All fixations were originating from this region and were ending in a specular vertical right-central sROI, this time encompassing the right eye. Scan paths in Group 2 exhibited a comparable vertical arrangement of eye movements, with fixations predominantly starting at a vertical left-central sROI before descending to the face midline encompassing nose and mouth regions yet including just the inner corner of the eyes. Group 2 displayed less fixation transitions across face regions, yet with a significantly higher number of fixations to the mouth region, when compared with the other two groups (as highlighted with iMap4 in Fig. 2). Lastly, Group 3 exhibited a more focal pattern of fixations, with marked eye-movement differences in both spatial and temporal dimensions. Fixations initiated at one small round sROI encircling the right eye, and then shifted with similar probability towards either the midline of the face or remained focused on the eyes. The next most likely transition in both cases was towards the eye region. Finally, a smaller proportion of fixations were redirected towards the face midline.

Our next objective was to assess whether idiosyncratic scanning patterns generalize across the 6 FEEs and the 2 stimulus modalities (12 conditions). We started by assessing how the 12 EM datasets of each observer distributed across the three identified face scanning strategies (Group 1, 2 or 3). This procedure allowed us to evaluate how consistent the observers were in their fixation strategy. This analysis revealed that 39% of observers consistently adhered to one single scanning strategy and had all twelve datasets of static and dynamic FEE clustering within one group. However, expecting observers to be consistent over 12 different conditions is a strict requirement, which overlooks the probability of random inconsistencies. Hence, adjusting the threshold to 10 stable conditions revealed that more than half of our subjects (60%) exhibited consistent sampling strategies. This stable use of a single fixation pattern across facial expressions and stimulus modality at the individual level confirms and extends previous similar findings, which were however only reported at the group level^12,20,24,38^. Moreover, we noted that observers were equally distributed across the three groups, suggesting no direct link between a specific pattern of fixation and the frequency of its usage.

Following up on these results, we then questioned whether any observed shift in strategy was driven by any specific FEE or stimulus modality (static vs. dynamic). Concerning stimulus modality, our data confirmed the consistent use of a single scanning strategy across both static and dynamic stimuli for most observers, suggesting that stimulus modality does not significantly impact visual strategies during FER. This contradicts previous findings reporting stable central fixations for dynamic FER compared to distributed fixation for static^24^. Potential explanations for this discrepancy might lie in some methodological differences between the two studies. First, in our study, we used face stimuli subtending a larger visual angle (14°) to elicit fixations on distinct facial features and better approximate the natural size of faces encountered during real-life social interactions. In contrast, the dynamic FEE used by Blais and colleagues^24^ subtended a smaller visual angle (5.72°). This might have allowed observers to sample most of the relevant facial information by only fixating the center of the faces, effectively reducing the need to visually explore the stimuli. Conversely, the larger visual angle used in the present study required observers to perform more fixations across the whole face in order to gather the visual information necessary for FER. Secondly, in the study conducted by Blais and colleagues^24^ stimulus duration was set to 500 ms. Under these time constraints, fixating towards the center of the face might have been more efficient for gathering information as quickly as possible, rather than tracking the different moving parts. Such a short presentation time limited the number of fixations, reducing the possibility of observers to fully explore the face stimuli. In contrast, we presented face stimuli for 1 s, closer to the duration of the natural unfolding of dynamic facial expressions. This longer and more ecological stimulus duration might have allowed our participants to sample visual information more closely as they were evolving over time. Taken together, our methodological choices revealed that observer’s idiosyncratic fixation patterns generalize across static and dynamic facial expressions, reflecting consistent scanning strategies independent of modality.

Concerning the impact of FEEs on scanning strategies, our data revealed that the strategy of Group 1 was significantly more frequently used to recognize “dynamic fear”, while Group 2’s sampling strategy was more often used to recognize “happiness”. These two observations suggest a potential link between particular emotions (happiness and dynamic fear) and the way observers allocate their attention to specific facial features during FER^38–42^. This idea is further supported by the differences in fixation locations between the two groups revealed by iMap4. Specifically, we found that Group 1 is characterized by a greater utilization of the eye region, which has been shown to elicit fixations to this region more frequently during the decoding of fear^39^. Additionally, allocating fixations to the eyes during the decoding of this FEE has been associated with enhanced classification accuracy^42,43^. On the other hand, the sampling strategy found in Group 2 focuses comparatively more on the mouth. Similarly, this is a diagnostic region that is sufficient, but also necessary, for the recognition of happiness^40,41^. No other FEE was statistically associated with any specific sampling pattern. This might suggest that for the decoding of the remaining FEEs, observers can efficiently gather relevant information by mainly using the same sampling strategy. Taken together, our findings support that each individual develops the most effective strategy that will benefit *them* in most situations, bypassing the need to constantly adapt to the presented stimulus.

Finally, we examined whether a specific sampling strategy was more effective than the others for FER performance. Our data revealed comparable scores of FER performance across the different groups for most FEE, except for the decoding of the dynamic expression of anger by observers in Group 2, who performed significantly better than those in Group 1. Fixation maps obtained for both groups suggest that observers who relied more on the mouth (Group 2) performed more accurately, which is in line with previous findings^21,42,43^.

## Conclusions

To conclude, our data revealed three distinct idiosyncratic visual strategies during FER by quantitatively measuring both spatial and temporal dimensions of eye fixations in a large group of healthy young adults. Those strategies are as effective to achieve FER and highly generalize across all six basic expressions and the static and dynamic modalities. These observations were established by using strong methodological approaches relying on robust data-driven analyses, coupled with ecologically valid static and dynamic FEE stimuli matching the visual angle and temporal duration of real-life interactions. The visual system information intake is not universal even for the biologically relevant recognition of FEEs. Individual differences are present in diverse face processing tasks and future research is necessary to clarify the cognitive and neurofunctional roots of these observations.

## Methods

### Participants

When assessing individual differences in sampling strategies during scene perception, Hsiao et al. (2021) observed a large effect size with 60 participants. To be precautious, we increased this number to 70. In the end, a total of 73 Western adult observers (12 males, age range 18–30 years, M = 21.53, SD = 3.03) recruited at the University of Fribourg (Switzerland) participated in this experiment. All participants had normal or corrected-to-normal vision with no history of neurological or psychiatric disorders. They were recruited at the University of Fribourg and received course credits for their participation. The study was approved by the ethical committee of the University of Fribourg. All experiments were performed in accordance with relevant guidelines and regulations. Informed consent was obtained from all participants before starting.

### Stimuli and procedure

**Figure 7:**
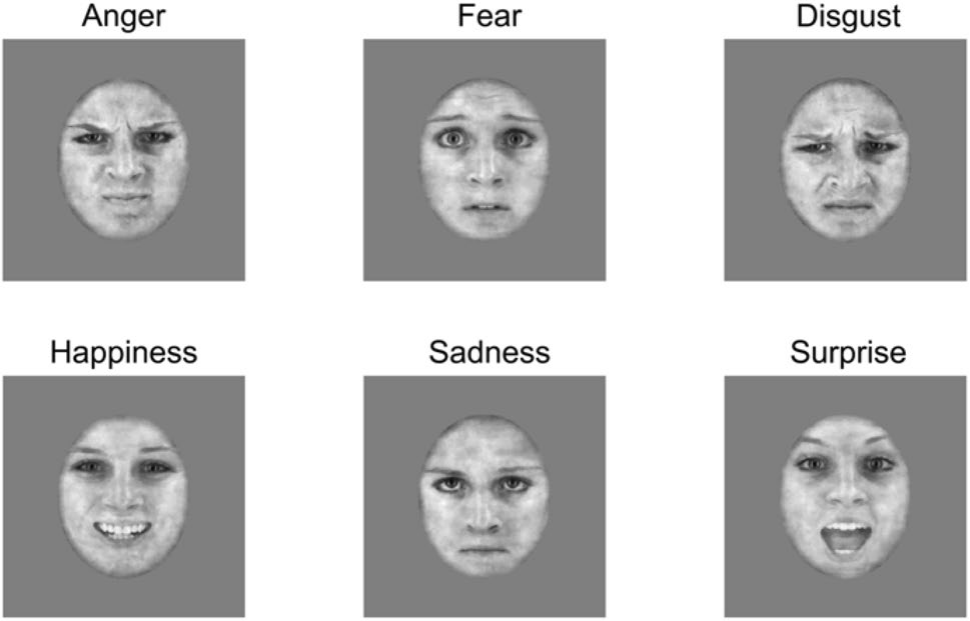
Illustration of the six static facial expressions of emotion for one female identity.

A total of 48 stimuli created by Gold and colleagues^28^ were used. The stimuli consisted of 4 female and 4 male identities, each portraying the six basic facial expressions of emotions (FEEs): anger, disgust, fear, happiness, sadness, and surprise (Ekman^1^). The stimuli consisted of either dynamic or static versions of each expression. The dynamic stimuli evolved from a neutral to a fully articulated expression over the course of 30 frames, while the static stimuli showed 30 repetitions of the last frame of each dynamic sequence corresponding to the apex of each expression (Fig. 7). The stimuli were normalized for their contrast, luminance and amount of energy transmitted over presentation time using the SHINE Toolbox^44^. Finally, we added visual noise to each frame of the static sequences to match the luminance and contrast that were present in each frame of the dynamic stimuli. Both procedures are detailed in a recent work by Richoz and colleagues^45^. Stimuli subtended 13.84° height × 11.02° width of visual angle at a distance of 70 cm from the screen and were shown on a VIEWPixx/3D screen with a resolution of 1920 × 1080 pixels.

**Figure 8:**
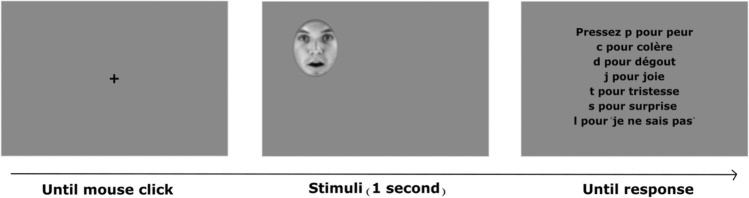
A schematic representation of the procedure. Each trial started with a central fixation cross followed by a facial expression presented for 1 s at a random location on the screen (e.g., top left). After each trial, participants provided their answer using labeled keys on a keyboard. The answer screen in French reads as follows: “press p for fear, c for anger, d for disgust, j for happiness, t for sadness, s for surprise, and I for ‘I don’t know’”.

The experiment was carried out using the Psychophysics^46,47^ and the EyeLink^48^ Toolboxes running on Matlab (R2014b, The MathWorks, Natick, MA). The oculomotor behavior of observers was recorded by tracking their left eye using an EyeLink 1000 Desktop Mount with a sampling rate of 1000 Hz. A nine-point calibration procedure was implemented before each testing session and repeated every 48 trials to ensure accurate gaze tracking. Each trial started with a fixation cross displayed at the center of the screen and participants were required to fixate it until it disappeared. This procedure was used to ensure the precision of calibration. Observers performed a total of 576 trials composed by 96 unique trials (6 FEEs × 8 identities × 2 modalities), each one repeated 6 times. On each trial, the face stimulus appeared in one of six randomized locations on the screen to reduce anticipatory strategies and ensure that the location of the first fixation was self-determined by the observer. Stimuli were presented for 1 s at a frequency rate of 30 Hz. This time constraint was used to prevent observers from adopting random sampling strategies after facial expression recognition and ensure a higher ecological validity of the recorded eye-movement data. Participants were instructed to freely explore each face and judge the facial expression portrayed. Following a 1-s presentation of the face stimulus, a list of all six expressions appeared on the screen. Participants used corresponding keyboard keys (i.e., dedicated keys letters for each expression) to select the perceived expression from this list (Fig. 8). The response window remained on the screen until a selection was made. Note that participants had also the option to choose a key labeled "I don't know" if they did not have enough time to see a given expression or for unknown answers.

### Statistical analysis

#### Preprocessing for data analysis

We applied the adaptive velocity algorithm developed by Nyström and Holmqvist^49^ to extract fixations. These were then realigned to a normalized space using iTemplate toolbox^50^. Finally, we filtered the data based on trial accuracy as further analysis will focus on fixations performed during correct trials only.

#### EMHMM

To explore eye movements data and quantitatively evaluate differences and similarities among individuals, we used the EMHMM toolbox^51^ which employs hidden Markov models (HMM). HMM capture in a compact fashion both spatial and temporal components of gaze behavior, proving to be particularly useful to analyze scan path on faces^52–55^. We started by providing the EMHMM algorithm with a total of 876 eye-movement (EM) datasets (73 participants × 12 conditions; Supplementary Fig. 2). Then, we used a variational Bayesian expectation maximization algorithm to estimate one HMM for each one of the 876 EM datasets, by initializing the seed random number generator with the 1000 value. To obtain each model, the algorithm determined the optimal number of hidden states within a predefined range from 1 to 3. In this context, hidden states correspond to statistical regions of interest (sROIs) on the face stimulus and the optimal number of sROI corresponds to the solution that maximizes the log-likelihood of each model. These sROI are represented by ellipses in HMM models. Subsequently, we used a variational hierarchical expectation maximization algorithm to cluster individual models together, by initializing the seed random number generator with the 1001 value. In this case, we used a fixed predefined number of hidden states corresponding to the median number of ROIs observed across the 876 models. To determine how many significantly different groups existed within our dataset, we adopted the following approach. We started by clustering individual models into 2 groups, which were then compared statistically. If they resulted significantly different, we increased the number of groups and repeated the comparison. This cycle was iterated until the resulting groups were no longer different. Finally, we determined, for each of the 876 EM to which group they were allocated. Using this information, we then explored scanning pattern consistency between modalities, FEEs and within subjects. For example, if the fixation patterns of subject A elicited while viewing static and dynamic FEEs were assigned to the same cluster, we could infer that the modality of presentation does not have a significant modulation effect on sampling strategies.

#### Fixation map analysis

We used the iMap4^23^ toolbox to statistically assess differences between the groups previously obtained using the EMHMM toolbox. Specifically, here we aimed to explore the representative fixation patterns obtained using EMHMM in terms of spatial information only. EMHMM considers number of fixations for spatial analyses, therefore we used the same measure when comparing the clusters in this section. iMap4 uses data-driven methods to assess statistical differences in terms of fixation distribution, without taking into consideration their temporal relationship. Fixation maps were smoothed using a two-dimensional Gaussian kernel function at 1° of visual angle by selecting the estimated option. This method consists in computing for each condition and observer, the expected values across trials. Finally, we normalized the maps by dividing them by the number of fixations performed in each trial. A pixel-wise linear mixed model was then applied on the smoothed normalized fixation maps and a multiple comparison correction was conducted by using a bootstrap spatial clustering method to control for type 1 errors. Fixation map analysis subsequently comparing the varying identified groups together involved number of fixations as response variable and participants as random predictors to account for the dependency.

#### Behavioral analysis

We used the unequal variances Mann–Whitney *U*-test to examine accuracy variations between groups while considering both expression and modality as factors. This involved comparing performance for one expression in one modality (e.g., Dynamic Anger) at a time between the three groups (3 comparisons each time). We corrected for multiple comparisons by dividing the significance level α = 0.05 by 3. All models were fitted in R 4.2.2^56^.

### Supplementary Information

Supplementary Figures.

## Data Availability

Informed consent was obtained from all participants for publication of identifying information in an online open-access publication. The data are available on the Open Science Framework at this link: https://osf.io/yc56w/?view_only=00c82ccf45d7468ba52168c1c386b850.

## References

[1] Ekman P, Friesen WV (1975). Unmasking the Face: A Guide to Recognizing Emotions from Facial Clues.

[2] Izard, C. E. *The face of Emotion* (Appleton-Century-Crofts, 1971).

[3] Darwin C (1872). The Expression of the Emotions in Man and Animals.

[4] Yitzhak N, Pertzov Y, Aviezer H (2021). The elusive link between eye-movement patterns and facial expression recognition. Soc. Personal Psychol. Compass.

[5] White D, Burton AM (2022). Individual differences and the multidimensional nature of face perception. Nat. Rev. Psychol..

[6] Blais C, Caldara R, Joan Y (2021). Culture Shapes Face Processing. Oxford Handbook of Cultural Neuroscience and Global Mental Health.

[7] Blais C, Jack RE, Scheepers C, Fiset D, Caldara R (2008). Culture shapes how we look at faces. PLoS ONE.

[8] Caldara R, Miellet S (2011). iMap: A novel method for statistical fixation mapping of eye movement data. Behav. Res. Methods.

[9] Caldara R (2017). Culture Reveals a Flexible System for Face Processing. Curr. Dir. Psychol. Sci..

[10] Yuki M, Maddux WW, Masuda T (2007). Are the windows to the soul the same in the East and West? Cultural differences in using the eyes and mouth as cues to recognize emotions in Japan and the United States. J. Exp. Soc. Psychol..

[11] Masuda T (2008). Placing the Face in Context: Cultural Differences in the Perception of Facial Emotion. J. Pers. Soc. Psychol..

[12] Jack RE, Blais C, Scheepers C, Schyns PG, Caldara R (2009). Cultural Confusions Show that Facial Expressions Are Not Universal. Curr. Biol..

[13] Gendron M, Roberson D, Van der Vyver JM, Feldman Barret L (2014). Perceptions of emotion from facial expressions are not culturally universal: evidence from a remote culture. Emotion.

[14] Jack RE, Garrod OGB, Yu H, Caldara R, Schyns PG (2012). Facial expressions of emotion are not culturally universal. Proc. Natl. Acad. Sci. U S A.

[15] Geangu E (2016). Culture shapes 7-month-olds’ perceptual strategies in discriminating facial expressions of emotion. Curr. Biol..

[16] Quesque F (2022). Does Culture Shape Our Understanding of Others’ Thoughts and Emotions? An Investigation Across 12 Countries. Neuropsychology.

[17] Stacchi L, Ramon M, Lao J, Caldara R (2019). Neural representations of faces are tuned to eye movements. J. Neurosci..

[18] Mehoudar E, Arizpe J, Baker CI, Yovel G (2014). Faces in the eye of the beholder: Unique and stable eye scanning patterns of individual observers. J. Vis..

[19] Arizpe J, Walsh V, Yovel G, Baker CI (2017). The categories, frequencies, and stability of idiosyncratic eye-movement patterns to faces. Vis. Res..

[20] Or CCF, Peterson MF, Eckstein MP (2015). Initial eye movements during face identification are optimal and similar across cultures. J. Vis..

[21] Yitzhak N, Pertzov Y, Guy N, Aviezer H (2022). Many Ways to See Your Feelings: Successful Facial Expression Recognition Occurs With Diverse Patterns of Fixation Distributions. Emotion.

[22] Hsiao JH, Lan H, Zheng Y, Chan AB (2021). Eye movement analysis with hidden Markov models (EMHMM) with co-clustering. Behav. Res. Methods.

[23] Lao J, Miellet S, Pernet C, Sokhn N, Caldara R (2017). iMap4: An open source toolbox for the statistical fixation mapping of eye movement data with linear mixed modeling. Behav. Res. Methods.

[24] Blais C, Fiset D, Roy C, Régimbald CS, Gosselin F (2017). Eye fixation patterns for categorizing static and dynamic facial Expressions. Emotion.

[25] Blais C, Roy C, Fiset D, Arguin M, Gosselin F (2012). The eyes are not the window to basic emotions. Neuropsychologia.

[26] Calvo MG, Nummenmaa L (2016). Perceptual and affective mechanisms in facial expression recognition: An integrative review. Cogn. Emot..

[27] Fiorentini C, Viviani P (2011). Is there a dynamic advantage for facial expressions?. J. Vis..

[28] Gold JM (2013). The Efficiency of Dynamic and Static Facial Expression Recognition. J. Vis..

[29] Bernstein M, Yovel G (2015). Two neural pathways of face processing: A critical evaluation of current models. Neurosci. Biobehav. Rev..

[30] Duchaine B, Yovel G (2015). A Revised Neural Framework for Face Processing. Annu. Rev. Vis. Sci..

[31] Hoffmann H, Traue HC, Bachmayr F, Kessler H (2010). Perceived realism of dynamic facial expressions of emotion: Optimal durations for the presentation of emotional onsets and offsets. Cogn. Emot..

[32] Wingenbach TSH, Ashwin C, Brosnan M (2016). Validation of the Amsterdam Dynamic Facial Expression Set ’ Bath Intensity Variations (ADFES-BIV): A Set of Videos Expressing Low, Intermediate, and High Intensity Emotions. PLoS One.

[33] Binetti N (2022). Genetic algorithms reveal profound individual differences in emotion recognition. Proc. Natl. Acad. Sci..

[34] de Lissa P (2021). Rapid saccadic categorization of other-race faces. J. Vis..

[35] Coutrot A, Hsiao JH, Chan AB (2018). Scanpath modeling and classification with hidden Markov models. Behav. Res. Methods.

[36] Hsiao JH, An J, Zheng Y, Chan AB (2021). Do portrait artists have enhanced face processing abilities? Evidence from hidden Markov modeling of eye movements. Cognition.

[37] Rodger H, Sokhn N, Lao J, Liu Y, Caldara R (2023). Developmental eye movement strategies for decoding facial expressions of emotion. J. Exp. Child Psychol..

[38] Vaidya AR, Jin C, Fellows LK (2014). Eye spy : The predictive value of fixation patterns in detecting subtle and extreme emotions from faces. Cognition.

[39] Smith ML, Cottrell GW, Gosselin F, Schyns PG (2005). Transmitting and decoding facial expressions. Psychol. Sci..

[40] Nusseck M, Cunningham DW, Wallraven C, Bülthoff HH (2008). The contribution of different facial regions to the recognition of conversational expressions. J. Vis..

[41] Beaudry O, Roy-Charland A, Perron M, Cormier I, Tapp R (2014). Featural processing in recognition of emotional facial expressions. Cogn. Emot..

[42] Schurgin MW (2014). Eye movements during emotion recognition in faces. J. Vis..

[43] Calvo MG, Fernández-Martín A, Gutiérrez-García A, Lundqvist D (2018). Selective eye fixations on diagnostic face regions of dynamic emotional expressions: KDEF-dyn database. Sci. Rep..

[44] Willenbockel V (2010). Controlling low-level image properties: The SHINE toolbox. Behav. Res. Methods.

[45] Richoz AR, Lao J, Pascalis O, Caldara R (2018). Tracking the recognition of static and dynamic facial expressions of emotion across the life span. J. Vis..

[46] Brainard DH (1997). The Psychophysics Toolbox. Spat. Vi..

[47] Kleiner M (2007). What’s new in psychtoolbox-3. Perception.

[48] Cornelissen FW, Peters EM, Palmer J (2002). The Eyelink Toolbox: Eye tracking with MATLAB and the Psychophysics Toolbox. Behav. Res. Methods Instr. Comput..

[49] Nyström M, Holmqvist K (2010). An adaptive algorithm for fixation, saccade, and glissade detection in eyetracking data. Behav. Res. Methods.

[50] Xiao NG, Lee K (2018). iTemplate: A template-based eye movement data analysis approach. Behav. Res. Methods.

[51] Chuk T, Chan AB, Hsiao JH (2014). Understanding eye movements in face recognition using hidden Markov models. J. Vis..

[52] Kanan C, Bseiso DNF, Ray NA, Hsiao JH, Cottrell GW (2015). Humans have idiosyncratic and task-specific scanpaths for judging faces. Vis. Res..

[53] Coutrot A, Binetti N, Harrison C, Mareschal I, Johnston A (2016). Face exploration dynamics differentiate men and women. J. Vis..

[54] Chan CYH, Chan AB, Lee TMC, Hsiao JH (2018). Eye-movement patterns in face recognition are associated with cognitive decline in older adults. Psychon. Bull. Rev..

[55] Zhang J, Chan AB, Lau EYY, Hsiao JH (2019). Individuals with insomnia misrecognize angry faces as fearful faces while missing the eyes: An eye-tracking study. Sleep.

[56] R: A Language and Environment for Statistical Computing. Preprint at (2022).

