# Supplementary material for: Idiosyncratic fixation patterns generalize across dynamic and static facial expression recognition
Source: Sci Rep. 2024 Jul 13;14:16193. doi: 10.1038/s41598-024-66619-4 (PMC11246522; doi:10.1038/s41598-024-66619-4)
Supplement: Supplementary file 1 — Supplementary Figures. [file 41598_2024_66619_MOESM1_ESM.docx]

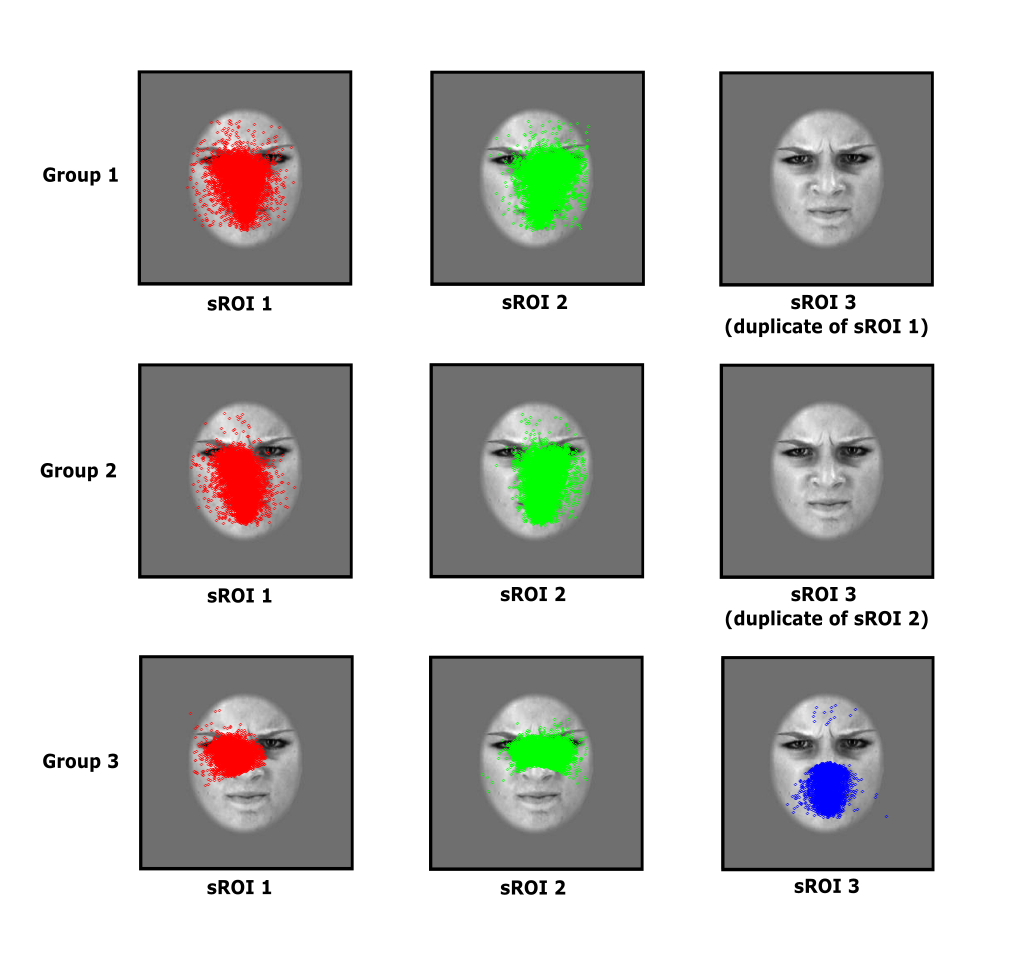


**Supplementary Figure 1.** Fixation distributions resulting from the EMHMM clustering procedure for the three groups of observers during FER. In Group 1 and 2, sROI 1 and 3 and sROI 2 and 3 are respectively duplicates of each other in that they cover nearly an identical spatial region. This sROI duplication is caused by specifying three sROIs when only two naturally exist. The three sROIs are required to fully account for the data of Group 3 because currently the EMHMM clustering procedure we used does not support an unequal number of sROIs between groups currently.

**Within Group 1**, in details, when computing the transition matrix during the estimation of the group HMMs, the algorithm randomly assigns the first (initial) sROI of a subject’s HMM to one of the two sROIs duplicate (i.e., 1 and 3), based on random initialization of the clustering algorithm. As a result, the first sROI of some subjects will map to sROI 1, and others will map to sROI 3, spreading prior probabilities of Group 1 HMM between sROI 1 and sROI 3. However, when inferring and plotting the *most likely* sROI sequence for a given fixation sequence, the (first) fixation is assigned to the sROI with the highest probability, since duplicates sROI1 and sROI 3 cover the same spatial region (and thus have the same emission likelihood). Here, sROI 1 has the higher prior probability of 65% compared to 35% of ROI 3. Thus, for Group 1, we observe no fixations in sROI 3 when plotting the most likely sROI sequences.

An analogous situation occurs **within Group 2** where the sROI 3 is a duplicate of sROI 2. Here, the transition matrix starts from sROI 1. When it next moves to the consecutive fixation, it randomly assigns the consecutive sROI of the subject’s HMM to one of the two identical sROIs, splitting the transition probability between sROI 2 and 3. As for Group 1, when plotting the *most likely* sROI sequence, fixations are assigned to the most likely sROI since the two sROIs cover the same spatial region. Here, sROI 2 has a higher transition probability of 60% compared to 40% of sROI 3. Thus again, for Group 2, we observe no fixations in sROI 3 when plotting the most likely sROI sequences.

Overall, this means that Group 1 and 2 are fully described by 2 sROIs, even though three ROIs were specified.

Therefore, the most appropriate formal reading of the transition matrices of Group 1 and 2 is to collapse sROIs 1 and 3, and sROIs 2 and 3 respectively, by summing up their probabilities.


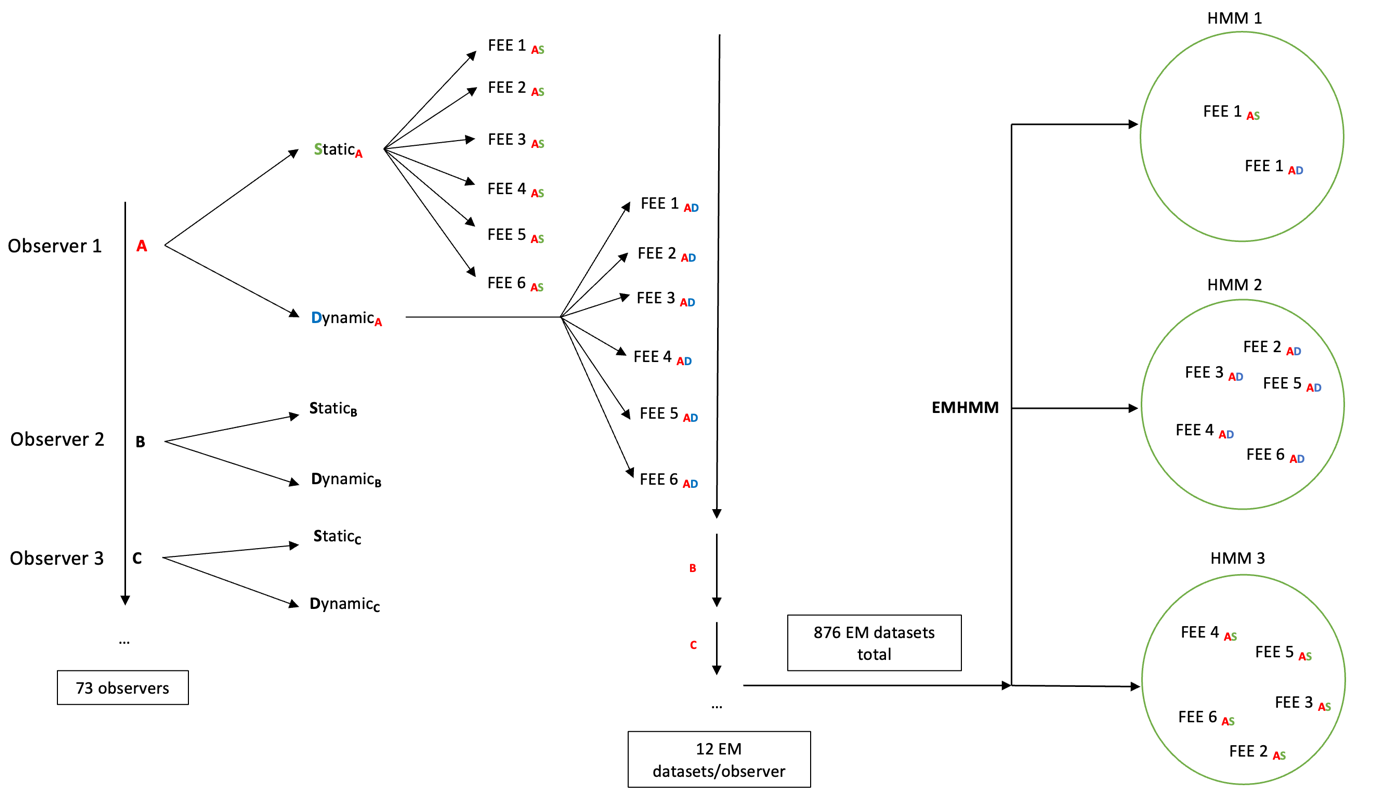


**Supplementary Figure 2**. Experimental design. 876 eye-movement (EM) datasets were used as input for the EMHMM algorithm. These corresponded to one EM dataset for each of the 73 participant in each one of the 12 experimental conditions (6FEEs x 2 modalities).
